# Prediction of tannins as a phytogenic additive effect on *in vitro* enteric methane emissions in ruminants: A meta-analysis

**DOI:** 10.5455/javar.2026.m1046

**Published:** 2026-06-22

**Authors:** Ana Karen Frutis-Moto, Gregorio Álvarez-Fuentes, Mendoza D. German, César Arturo Ilizaliturri-Hernández, Luis Octavio Negrete-Sanchez, Alfonso Chay-Canul, Héctor Aarón Lee-Rangel

**Affiliations:** 1 Posgrado Multidisciplinario en Ciencias Ambientales, Universidad Autónoma de San Luis Potosí, San Luis Potosí 78210, México; 2 Facultad de Agronomía y Veterinaria, Centro de Biociencias, Universidad Autónoma de San Luis Potosí, San Luis Potosí 78321, México; 3 Departamento de Producción Agrícola y Animal, Universidad Autónoma Metropolitana–Xochimilco, Mexico City CP 04960, México; 4 Coordinación para la Innovación y Aplicación de la Ciencia y la Tecnología (CIACYT), Universidad Autónoma de San Luis Potosí, Av. Sierra Leona núm. 550-2ª, Lomas de San Luis, San Luis Potosí, C.P. 78210, México; 5 División Académica de Ciencias Agropecuarias, Universidad Juárez Autónoma de Tabasco, Carr. Villahermosa-Teapa, km 25, Villahermosa 86280, México

**Keywords:** Condensed tannins, *in vitro* methane emissions, meta-analysis, prediction equation, ruminal fermentation, ruminal digestibility

## Abstract

**Objectives:** This study aimed to quantify the effects of dietary tannins on *in vitro* methane emissions and to develop substrate-specific predictive equations for methane estimation in ruminants.

**Materials and Methods:** A systematic review was conducted following PRISMA guidelines, including studies published between 2010 and 2024 in Scopus, Web of Science, PubMed, and ScienceDirect. A total of 32 studies (213 observations) met the inclusion criteria and were classified into foliage (*n* = 130) and forage (*n* = 83) datasets. Pearson correlations and stepwise regression models were developed using 75% of the data and validated with the remaining 25%.

**Results:** Tannin concentrations showed high variability (0.40–391.00 gm/kg DM). In foliage, methane emissions were negatively correlated with condensed tannins (*r* = –0.40; *p* < 0.001), whereas in forages, total tannins showed a positive correlation (*r* = 0.27; *p* < 0.05). Predictive models demonstrated moderate accuracy, with higher performance in forage (R² = 54.61%; RMSEP = 0.13) compared to foliage (R² = 28.45%; RMSEP = 0.53). These contrasting responses highlight a strong substrate-dependent effect of tannins on methanogenesis.

**Conclusions:** Methane mitigation is influenced not only by tannin concentration, particularly condensed tannins, but also by substrate type and chemical composition. This study provides a novel substrate-specific predictive framework integrating tannin fractions and bromatological variables, offering a practical tool for rapid screening of antimethanogenic feed resources. However, model accuracy could be improved by incorporating physiological and fermentation-related variables.

## 1. Introduction

The increase in global food demand has driven the intensification of livestock systems; as a result, global meat and milk production have risen by 6% and 10%, respectively, over the past five years [1]. This increase has contributed to rising greenhouse gas emissions, particularly methane, a major contributor to global warming. It is estimated that this gas has a global warming potential 28 times greater than that of carbon dioxide [2], with an atmospheric lifetime of 12.4 years [3]. Its production is primarily associated with anthropogenic activities, notably the agricultural sector, especially ruminal fermentation and manure management [4], which together account for approximately 32% of global methane emissions [5]. In addition to being an environmental problem, methane production results in an energy loss ranging from 7% to 12% [6], affecting growth and productive development [7]. For this reason, various strategies aimed at reducing methane emissions and improving the efficiency of metabolic energy utilization have been evaluated, notably the use of phytogenic additives such as tannins.

The inclusion of tannins in animal diets has been extensively investigated in multiple studies, which have examined different doses and substrate types [8, 9, 10]. Tannins are phenolic compounds classified as condensed tannins (CT) and hydrolyzable tannins (HT) [11]. CTs are flavan-3-ol subunits linked together to form oligomers and polymers, while HTs are esters of gallic or ellagic acid [12]. These compounds directly influence ruminal fermentation by modifying microbial populations, particularly protozoa and cellulolytic bacteria such as Ruminococcus albus, Ruminococcus flavefaciens, and Ruminococcus amylophilus, which are responsible for fermenting fiber and starch into volatile fatty acids, generating hydrogen as a byproduct [13, 14]. This hydrogen is subsequently utilized by methanogenic archaea to reduce carbon dioxide and produce methane [15]. Likewise, tannins may promote populations of *Fibrobacter succinogenes* [16], which has been associated with reduced hydrogen production and lower protozoal activity [17]. However, their effects are not always linear, as high doses may impair digestibility and feed intake [18]. In the context of studying these processes, mathematical models serve as representations of complex biological systems [19] and describe biological phenomena through equations [20]. Although several meta-analyses have evaluated the effects of tannins on ruminal methanogenesis [21, 22, 23], current evidence remains inconsistent and difficult to generalize due to high variability in tannin type, concentration, and substrate composition. Most previous studies have focused on isolated effects of tannins without adequately accounting for the interaction between tannin fractions and the chemical characteristics of the substrate. Additionally, existing approaches have not developed substrate-specific predictive models capable of distinguishing between forage- and foliage-based systems, limiting the accuracy and applicability of methane prediction, particularly under *in vitro* conditions where variability is inherently high. Therefore, there is a need for an integrative framework that combines tannin fractions with bromatological variables and explicitly considers substrate type to improve the prediction and understanding of methane emissions in ruminants. In this context, the objective of this study was to evaluate the effects of tannins on *in vitro* methane emissions and to develop substrate-specific predictive equations by integrating tannin fractions and bromatological variables.

## 2. Materials and Methods

### 2.1. Ethical approval

As a meta-analysis, this study does not require ethical approval.

### 2.2. Data search

A literature search was conducted in accordance with the PRISMA (Preferred Reporting Items for Systematic Reviews and Meta-Analyses) guidelines [24]. The search included studies published between January 2010 and December 2024 in Scopus (Elsevier, https://www.scopus.com/), Web of Science (Clarivate, https://www.webofscience.com/), PubMed (National Institutes of Health, https://pubmed.ncbi.nlm.nih.gov/), and ScienceDirect (Elsevier, https://www.sciencedirect.com/) databases. Boolean operators (“OR” and “AND”) were used to combine the search terms as follows: (“ruminant” OR “bovine” OR “cattle” OR “cow” OR “sheep” OR “ovine” OR “heifer” OR “calf” OR “lambs” OR “goat” OR “caprine”) AND (“natural additives” OR “feed additives” OR “natural extracts” OR “tannins”) AND (“methane” OR “enteric methane” OR “fermentation”).

**Figure 1.**
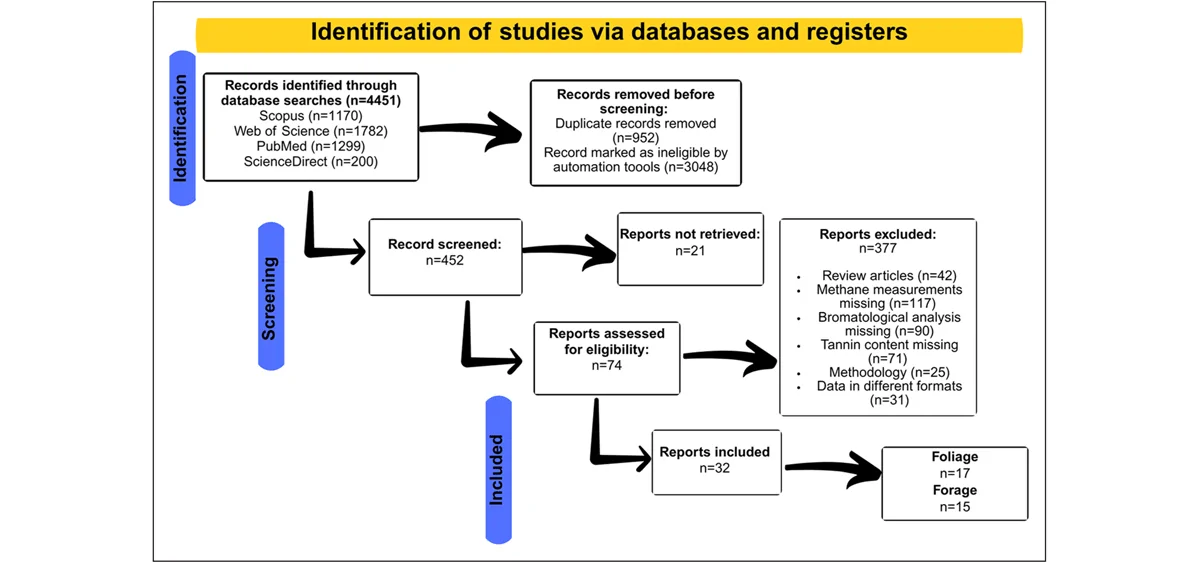
Flowchart of the study identification, screening, eligibility, and inclusion process according to PRISMA.

The initial search yielded 4,451 articles. After removing 952 duplicates, 3,499 records remained for screening. Title and abstract screening excluded 3,048 studies that did not meet the inclusion criteria. The full text of the remaining 452 articles was assessed for eligibility; 420 were excluded according to the predefined criteria (detailed below). The final dataset comprised 32 publications (Figure 1).

### 2.3. Inclusion and exclusion criteria

Studies were included if they met the following criteria: (i) *in vitro* assays using bovine or ovine ruminal fluid; (ii) methane measurement using gas production techniques (syringe-based methods or gas chromatography) at 24 h of incubation; (iii) methane values reported as ml/gm of dry matter (DM); (iv) specification of substrate type and complete chemical composition; and (v) reporting of tannin-related variables, including total tannins (TT), phenolic tannins (FT), hydrolyzable tannins (HT), and condensed tannins (CT).

Studies were excluded if methane was reported only in figures or using units not convertible to the standardized format, if substrate chemical composition was not fully reported, or if the substrate was not of forage origin. In addition, studies including polyethylene glycol (PEG) were excluded because PEG can neutralize tannins and therefore confound their effects on methane emissions [23].

### 2.4. Data extraction

From each of the 32 selected articles, which together contributed 213 observations to this meta-analysis database, bibliographic information (authors, year, DOI), substrate type and characteristics, substrate chemical composition, tannin-related variables, and methane measurements (reported as ml/gm DM or ml/200 gm DM) were extracted. All bromatological variables were standardized to gm/kg DM, and methane values were standardized to ml/gm DM. Tables 1 and 2 summarize the included studies and tannin contents used in the present analysis. The database was classified into two categories based on substrate origin: foliage (17 articles; 130 observations) and forage (15 articles; 83 observations). For each study, the mean, standard deviation (SD), and sample size (n)—corresponding to the number of *in vitro* incubations—were extracted. In cases where the standard deviation was not reported, it was estimated from the standard error of the mean (SEM) multiplied by the square root of the sample size, using the equation SD = SE × √n [22]. Prior to analysis, all data were transformed using the natural logarithm (ln) to stabilize the variance.

**Table 1.** Experiments included modeling, levels of tannins and methane in foliage.

Exp no.	Tannin source	Total phenols(gm/kg)	Total tannin phenols (gm/kg)	Condensed tannins (gm/kg)	Hydrolysable tannins (gm/kg)	Methane(ml/kg) DM	Reference
1	*Acacia nilotica*	163	148	7.9	140.10	12.7	Aderao et al. [25]
	*Ziziphus nummularia* (leaves)	16.3–52.5	9.40–31.1	2.3–28.3	1.20–38.10	23–34	
2	Various	11.00–211.00	7.13–190	1.15–50.40	5.58–166.00	5.6–27.6	Baruah et al. [26]
3	Various	40.30–312.00	2.50–29.20	0.2–23.20	2.47–6.02	1.55–28.7	Bhatta et al. [27]
4	Various	11.50–111.00	7.20–331.60	0.01–260.00	0.71–103.00	0.43–9.15	Bhatta et al. [28]
5	Various	16.00–274.00	8.00–262.00	0.10–82.00	5.00–252.00	6.04–27.26	Bhatta et al. [29]
6	*Acacia nilotica*	76.70	54.20	53.60	–	4.52	Geneviève et al. [30]
	*Acacia raddiana*	401.30	391.60	3.40	–	2.83	
7	Various	6.70–170.80	0.80–161.50	0.20–38.50	0.60–159.80	20.3–28.45	Jadhav et al. [31]
8	*Gleditsia triacanthos* (leaves)	–	–	154.00	–	5.48	Kaya et al. [32]
9	Various	0.59–0.80	0.40–0.54	0.12–0.68	–	20.73–25.75	Khelalfa et al. [33]
11	*Fraxinus angustifolia*	18.92	11.08	2.78	–	31.55	Mebirouk-Boudechiche et al. [34]
	*A. dealbata*	35.98	38.32	10.27	–	14.8	
12	Various	24.40–90.10	8.00–59.6	3.00–97.70	–	5.25–37.65	Melesse et al. [35]
13	Various	3.95–99.70	1.41–82.10	0.69–47.20	–	7.93–16.4	Pal et al. [36]
14	Various	–	–	112.00–361.00	9.00–350.00	1.66–3.54	Rira et al. [37]
15	Various	–	–	39.00–92.00	–	12.4–17.3	Rira et al. [38]
16	*Eucalyptus* (Various navels)	114.80–141.50	103.50–105.60	1.30–4.10	–	5.1–5.4	Sallam et al. [39]
17	Various	24.30–386.50	9.10–319.80	–	–	1.58–63.75	Theart et al. [40]

**Table 2.** Experiments included modeling, levels of tannins and methane in forage.

Expt no.	Tannin source	Total phenols(gm/kg) DM	Total tannin phenols(gm/kg)	Condensed tannins(gm/kg)	Hydrolysables tannins(gm/kg)	Methane(ml/kg) DM	Reference
1	*V. sinesis* (hay)	3.90	2.90	1.20	1.70	28.40	Aderao et al. [25]
2	*C. plectostachyus*	3.40	–	–	–	23.31	Ángeles-Mayorga et al. [41]
	*C. aconitifolius*	10.30	–	–	–	19.45	
3	*C. parasitica*	65.68	50.60	3.68	46.90	29.20	Baruah et al. [26]
	*L. linifolia*	22.00	12.50	0.24	12.30	27.00	
4	Various	10.80–185.00	0.60–16.91	0.00–18.70	0.29–16.90	7.50–41.80	Bhatta et al. [28]
5	Various	11.00–71.00	7.00–51.00	0.10–32.00	5.00–32.00	12.67–30.88	Bhatta et al. [29]
6	Various	13.00–23.10	–	22.50–136.00	7.80–37.00	24.20–26.50	Brinkhaus et al. [42]
7	Various	12.28–29.28	6.91–18.43	1.48–6.75	–	18.00–50.10	Mebirouk-Boudechiche et al. [34]
8	*S. sesban*	32.20	12.30	11.20	–	30.80	Melesse et al. [35]
	*C. palmensis*	82.20	33.40	0.10	–	27.30	
9	*Dalea purpurea*Various	28.60–66.20	–	78.90–84.50	–	26.00–27.30	Peng et al. [43]
10	Various	12.00–69.30	7.40–64.10	0.10–12.80	7.30–51.30	18.50–84.50	Rajkumar et al. [44]
11	*M. sativa*	10.20	6.60	0.20	–	11.50	Sallam et al. [39]
12	Various	6.00–51.70	6.60–34.80	0.20–23.20	–	41.40–57.40	Soltan et al. [45]
13	Various	44.90–189.30	28.20–148.30	–	–	42.00–64.00	Theart et al. [40]
14	*Avena sativa*	12.20	7.50	0.20	7.30	52.10	Leitanthem et al. [46]

### 2.5. Statistical analysis

All data were analyzed using SAS statistical software [47]. Data were extracted from each study. Descriptive statistics for the chemical composition variables were calculated using the PROC MEANS procedure. Pearson’s correlation coefficients were calculated using SAS PROC CORR to assess the relationships between the bromatological variables and methane production. Correlated explanatory variables (|*r*| > 0.7) were not included simultaneously in the model to avoid multicollinearity effects.

For normalization, variables were log_10_-transformed. Pearson correlation coefficients among explanatory variables were calculated using PROC CORR to explore variable relationships and to identify highly correlated predictors. Variables with strong correlations (|*r*| > 0.7) were not included simultaneously in the model to minimize multicollinearity.

Tests for heterogeneity and bias were performed using RStudio (version 4.5.2) and the “metafor” package [22]. Heterogeneity was assessed with chi-square (Q) tests and I^2^ statistic (interpreted as low, moderate, substantial, or high, per Deeks et al. [48]). Publication bias was examined with the funnel plot, Begg’s correlation, and Egger’s regression. Asymmetry was considered present when tests were significant (*p* < 0.10) [21].

Model development was conducted according to St-Pierre [49]. Briefly, 75% of the observations were randomly selected to build prediction models using the GLM and Stepwise procedures in SAS, applying the following general model:


\[
Y\; = \;{B_0}\; + \;{B_1}{X_1}\; + \;{B_3}{X_3}\;{ \ldots }\;{B_{15}}{X_{15}}\; + \;\varepsilon
\]

where *Y* is the response variable (methane emissions), *β* represents regression parameters, and *X* corresponds to the predictor (explanatory) variables. The remaining 25% of the data from each substrate category was used for model validation. Model performance was evaluated using *p*-values, mean square error of prediction (MSE), and root mean square error of prediction (RMSEP) as goodness-of-fit indicators.

## 3. Results and Discussion

### 3.1. Heterogeneity and Bias

The bias analyses reported in Table 3. Although significant heterogeneity was detected (Q test; *p* < 0.10), no evidence of publication bias was observed according to funnel plot symmetry and Begg’s and Egger’s tests given the significant heterogeneity reported (Q; *p* < 0.10) [22]. However, no publication bias was observed in either classification. In both cases, asymmetry appeared on the left side. For heterogeneity analyses (I^2^), forage showed a lower I² (75.26%), while foliage showed a higher one (58.99%). These data are consistent with those reported by [21, 50], indicating considerable and high heterogeneity in both cases. This suggests that heterogeneity is due to variability across studies rather than to a few influential factors, such as tannin type, *in vitro* assay, source type, or data quality [50].

**Table 3.** Heterogeneity and bias data from modeling

Classification	SE	95% CI		*p*-value	Q	Heterogeneity *p*-value	I^2^ (%)
		Lower	Upper				
Forage	0.0236	1.3328	1.4859	0.5030	323.9042	<0.001	75.26
Foliage	0.0317	0.9190	1.0300	0.7488	607.534	<0.001	58.99

SE = Standard Error; Q = chi-squared statistic and associated significance level (*p*-value); I^2^ = percentage of variation.

### 3.2. Tannin concentration

The tannin content (gm/kg DM) showed high variability in both substrate classifications (Tables 1, 2). In foliage, total tannins ranged from 0.40 to 391.00 gm/kg DM. According to Iqbal and Poór [51], tannin content can represent up to 25% of DM in the leaves, roots, and bark of woody species. Regarding condensed tannins, maximum values of 136.00 gm/kg DM were recorded, mainly in forages, which fall within the ranges described by Mueller-Harvey et al. [52], who documented concentrations of up to 200 gm/kg DM.

Tannin accumulation is regulated by both genetic and environmental factors, including soil type, plant nutritional status, and phenological stage [51, 53]. For example, Sakariyahu et al. [54] reported increases of up to 33% in total phenol concentration in low-fertility soils. In the present study, maximum total phenol concentrations of up to 391.00 gm/kg DM in forage and 148.30 gm/kg DM in foliage were observed, exceeding the maximum values reported by Rana et al. [55], who documented maximum values of 174.00 gm/kg DM. These differences suggest that environmental and/or edaphic conditions may have favored the synthesis and accumulation of tannins in the evaluated substrates.

Seasonality may also explain part of the observed variability. Rana et al. [55] noted that some species increase tannin synthesis during summer, whereas others show a reduction, even within the same species. Such fluctuations reflect the influence of climatic factors and plant defense mechanisms, contributing to the heterogeneity observed across studies.

From a nutritional standpoint, high tannin concentrations can have adverse effects on ruminants, including reduced nutrient utilization, decreased palatability, and consequently lower voluntary feed intake [56]. Rira et al. [57] reported that condensed tannin levels above 50.00 gm/kg DM reduce digestibility. Similarly, Sun et al. [18] documented reductions of up to 50% in digestibility when hydrolyzable tannins exceeded 6% of DM in sheep diets supplemented with chestnut extract.

### 3.3. Correlations and mechanisms of action

**Figure 2.**
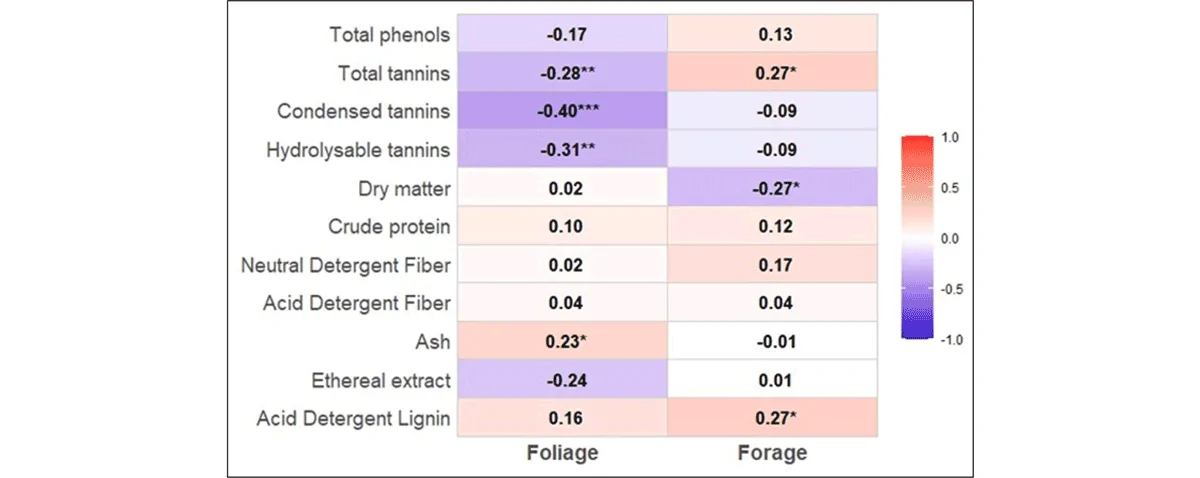
Correlations coefficients (*r*) between chemical composition and various tannin and methane parameters. *p < 0.05; **p < 0.01; ***p < 0.001.

Correlation analyses showed contrasting patterns between forage and foliage substrates (Figure 2). In foliage, total tannins showed a significant negative correlation with methane emissions (*r* = –0.28; *p* < 0.01), while condensed tannins exhibited the strongest association (*r* = –0.40; *p* < 0.001). Similarly, hydrolyzable tannins were negatively correlated with methane (*r* = –0.31; *p* < 0.01). These results are consistent with Pal et al. [36], who reported negative correlations for foliage with total tannins (*r* = –0.32; *p* < 0.05) and condensed tannins (*r* = –0.22; *p* < 0.05). Collectively, these findings suggest that both tannin origin and chemical structure may modulate the mechanisms involved in methane mitigation.

In contrast, forages exhibited a positive correlation between total tannins and methane (*r* = 0.27; *p* < 0.05), whereas correlations with condensed and hydrolyzable tannins were not significant. Previous studies have indicated that tannin effects depend on molecular weight, degree of polymerization, source type, and concentration [58, 59]. In addition, the presence of other secondary metabolites in foliage may alter microbial activity and fermentation patterns [60], which could partially explain the contrasting correlations observed between substrate categories.

Methane mitigation has been more strongly associated with condensed tannins (CT) than with hydrolyzable tannins (HT) [41]. This effect has been attributed to the formation of CT–protein complexes that limit ruminal degradation [61, 62], thereby reducing hydrogen availability for methane formation [21]. In contrast, HT may reduce methane emissions without markedly affecting degradability, potentially due to the presence of gallic acid derivatives [41]. Moreover, the combination of CT and HT may enhance antimethanogenic effects through synergistic interactions among phenolic compounds [21, 63].

Regarding fiber components, acid detergent lignin (ADL) showed a positive and significant correlation with methane emissions in forage (*r* = 0.27; *p* < 0.05). Changes in fiber fractions can either increase or reduce methane emissions [58]. According to Anantasook et al. [64], lignin may explain up to 90% of the variability in methane estimates. However, in highly lignified forages, increased lignin has often been associated with lower methanogenesis due to reduced nutrient availability and fermentability [58, 65]; in the present analysis, the positive correlation suggests that higher lignin levels may have limited organic matter digestibility, particularly within the neutral detergent fiber fraction, thereby promoting fermentation conditions in which methanogenic activity is not effectively inhibited [66, 67].

Both tannin and lignin contents have been associated with reduced ruminal microorganism populations, particularly protozoa, leading to lower methane emissions [68, 69]. Nevertheless, the magnitude of these effects depends on the interactions among phenolic compounds, fiber structure, and fermentation conditions.

### 3.4. Predictive equations

An equation was obtained for each classification (Table 4). For forage, the model included eight variables: three related to tannin content and five associated with bromatological characteristics. For foliage, the equation included four variables: one associated with tannins and three related to bromatological composition. The coefficient of determination was lower for foliage (R^2^ = 28.45%) compared with forage (R^2^ = 54.61%).

**Table 4.** Equations generated by Stepwise multiple linear regression of tannin and chemical composition on the determined methane.

Classification	Regression equations	R^2^ %	Adjusted R^2^ %	MSE	RMSE
Forage	9.38 – (0.05 × CT) – (0.50 × TP) + (0.35 × TT) – (1.54 × DM) – (2.88 × ADF) + (0.47 × ASH)	54.61	52.93	0.018	0.136
Foliage	–17.90 – (0.62 × TP) + (7.51 × DM) – (1.31 × CP) + (0.27 × ADF)	28.45	25.98	0.035	0.533

CT = Condensed Tannins; TP = Total phenols; TT = Total tannins; DM = Dry matter; ADF = Acid Detergent Fiber; CP = Crude protein; MSE = Mean squared prediction error; RMSE = Root mean square prediction error.

These values were lower than those reported by Jayanegara et al. [70], who developed regression equations with higher coefficients of determination (adjusted R² = 86.7%), although using fewer observations. The lower coefficients obtained in the present study may be related to the use of *in vitro* techniques, which have shown limited predictive accuracy, particularly in diets supplemented with tannins, where methane reduction may be linked to lower apparent organic matter digestion, especially of the fibrous fraction [23]. These methodological and biological sources of variability may increase dispersion in methane measurements and consequently reduce model accuracy.

The inclusion of crude protein and fiber fractions (NDF and ADF) confirms their relevance in methane emissions, consistent with previous modeling efforts [70]. Likewise, fiber fractions are strongly associated with dry matter content [65], a relationship observed in both equations. Additionally, it has been shown that using broader databases and incorporating variables with associative effects can improve goodness-of-fit indicators [71], as these factors may influence proteolysis mechanisms and, consequently, methane emissions, depending on tannin type and dietary proportion [72]. This could explain the behavior of the forage equation, as the inclusion of a higher number of predictors reduced the root mean square error of prediction (RMSEP = 0.13) compared with the foliage model (RMSEP = 0.53), suggesting stronger interactions between tannins and substrate chemical composition in forage-based datasets.

The moderate predictive performance obtained in both equations reflects the complexity of the mechanisms regulating methane emissions in ruminants, which are not determined exclusively by tannin or phenolic concentrations. The antimethanogenic response also depends on tannin chemical structure, degree of polymerization, and interactions with dietary components and the ruminal microbiota [23, 67]. It has also been reported that balanced diets with appropriate protein–energy ratios may reduce ruminal protein degradability and methane emissions [21].

Moreover, the absence of critical physiological variables—such as dry matter intake, passage rate, volatile fatty acid profiles, or methanogenic microbial abundance—may have limited model performance. Previous studies have shown that including parameters such as fiber digestibility, fermentation kinetics, and rumen ecosystem structure can substantially improve methane prediction accuracy [73, 74].

## 4. Conclusions

The present meta-analysis demonstrates that the antimethanogenic effect of tannins is strongly dependent on both their concentration and chemical nature, particularly condensed tannins, as well as on substrate type and composition. The contrasting responses observed between foliage and forage systems confirm that tannin–methane interactions are substrate-specific and should not be generalized across feed resources. The predictive models developed in this study provide a novel framework for estimating methane emissions based on tannin fractions and bromatological variables, with moderate predictive performance (R^2^ = 54.61% for forage and 28.45% for foliage). These findings advance current knowledge by highlighting the importance of integrating substrate classification into methane prediction models. From a practical perspective, the results support the strategic use of tannin-rich feed resources as a feasible approach for methane mitigation in ruminant production systems and offer a tool for the rapid screening of potential antimethanogenic substrates under *in vitro* conditions. However, the moderate predictive accuracy indicates that methane emissions are influenced by complex interactions beyond tannin content alone. Future research should incorporate physiological variables, rumen fermentation parameters, and microbial dynamics to improve model robustness and applicability under *in vivo* conditions.

## Data Availability

The data presented in this study are available from the corresponding author upon reasonable request.

## List of abbreviations

PRISMA, Preferred Reporting Items for Systematic Reviews and Meta-Analyses; DM, dry matter; TT, total tannin; FT, phenolic tannins; HT, hydrolyzable tannins; CT, condensed tannins; PEG, polyethylene glycol; GLM, general lineal model; MSE, mean square error of prediction; NDF, neutral detergent fiber; ADF, acid detergent fiber; RMSE, Root mean square prediction error.

## References

[1] FAO (2025). Sustainable food systems: concept and framework. https://www.fao.org/3/ca2079en/ca2079en.pdf.

[2] EPA (2025). Importance of Methane | US EPA. https://www.epa.gov/gmi/importance-methane.

[3] Myhre G, Shindell D, Bréon FM, Collins W, Fuglestvedt J, Huang J (2013).

[4] IPCC (2023).

[5] FAO (2025). Livestock and enteric methane. https://www.fao.org/in-action/enteric-methane/background/en.

[6] Xie X, Cao Y, Li Q, Li Q, Yang X, Wang R (2025). Mitigating enteric methane emissions: An overview of methanogenesis, inhibitors and future prospects. Anim Nutr.

[7] Berça AS, Cardoso ADS, Longhini VZ, Tedeschi LO, Boddey RM, Berndt A (2019). Methane production and nitrogen balance of dairy heifers grazing palisade grass cv. Marandu alone or with forage peanut. J Anim Sci.

[8] Van Mullem J, Jeyanathan J, Peiren N, Vermeir P, Valckx J, Govaerts W (2025). Screening of and mechanistic insights into the enteric methane mitigation potential of European native and non-native forage trees, shrubs, and herbs using *in vitro* batch culture. Grass Forage Sci.

[9] Alecrim FB, Devincenzi T, Reyno R, Mederos A, Zinno CS, Mariotta J (2024). Addition of tannin-containing legumes to native grasslands: Effects on enteric methane emissions, nitrogen losses and animal performance of beef cattle. Sustainability.

[10] Gonzalez-Ronquillo M, Ghavipanje N, Jimenez LER, Cardoso-Gutiérrez E, Moreno JMP, Renna M (2025). *In vitro* assessment of dietary mealworm (*Tenebrio molitor* L.) combined with a natural source of tannins (*Acacia farnesiana* L.) for sheep feeding. Heliyon.

[11] Wang M, Yang T, Wang R, Fang X, Zheng J, Zhao J (2025). Ruminant methane mitigation: Microbiological mechanisms and integrated strategies for sustainable livestock production in the context of climate change. Renew Sustain Energy Rev.

[12] Naumann HD, Tedeschi LO, Zeller WE, Huntley NF (2017). The role of condensed tannins in ruminant animal production: Advances, limitations and future directions. Rev Bras Zootec.

[13] Abarghuei MJ, Boostani A (2025). Investigating the use of *Chenopodium quinoa* to improve rumen biofermentability and reduction of methane and carbon dioxide production. Vet Anim Sci.

[14] Zhao R, Sun J, Lin Y, Yan H, Zhang S, Huo W (2025). Effects of dietary tannic acid and tea polyphenol supplementation on rumen fermentation, methane emissions, milk protein synthesis and microbiota in cows. Microorganisms.

[15] Mizrahi I, Jami E (2018). Review: The compositional variation of the rumen microbiome and its effect on host performance and methane emission. Animal.

[16] Chen L, Bao X, Guo G, Huo W, Xu Q, Wang C (2021). Effects of hydrolysable tannin with or without condensed tannin on alfalfa silage fermentation characteristics and in vitro ruminal methane production, fermentation patterns, and microbiota. Animals.

[17] Bhatt RS, Sarkar S, Sharma P, Soni L, Sahoo A (2023). Comparing the efficacy of forage combinations with different hydrolysable and condensed tannin levels to improve production and lower methane emission in finisher lambs. Small Ruminant Res.

[18] Sun M, Liu P, Xing Y, Zhang M, Yu Y, Wang W (2025). Effects of chestnut tannin on nutrient digestibility, ruminal protease enzymes, and ruminal microbial community composition of sheep. Fermentation.

[19] Tedeschi LO (2006). Assessment of the adequacy of mathematical models. Agric Syst.

[20] Osorio AI, Mendoza GD, Plata FX, Martínez JA, Vargas L, Ortega GC (2015). A simulation model to predict body weight gain in lambs fed high-grain diets. Small Ruminant Res.

[21] Brutti DD, Canozzi MEA, Sartori ED, Colombatto D, Barcellos JOJ (2023). Effects of the use of tannins on the ruminal fermentation of cattle: A meta-analysis and meta-regression. Anim Feed Sci Technol.

[22] Orzuna-Orzuna JF, Dorantes-Iturbide G, Lara-Bueno A, Mendoza-Martínez GD, Miranda-Romero LA, Hernández-García PA (2021). Effects of dietary tannins’ supplementation on growth performance, rumen fermentation, and enteric methane emissions in beef cattle: A meta-analysis. Sustainability.

[23] Jayanegara A, Leiber F, Kreuzer M (2012). Meta-analysis of the relationship between dietary tannin level and methane formation in ruminants from *in vivo* and *in vitro* experiments. J Anim Physiol Anim Nutr.

[24] Page MJ, McKenzie JE, Bossuyt PM, Boutron I, Hoffmann TC, Mulrow CD (2021). The PRISMA 2020 statement: An updated guideline for reporting systematic reviews. BMJ.

[25] Aderao GN, Sahoo A, Bhatt RS, Kumawat PK, Soni L (2018). *In vitro* rumen fermentation kinetics, metabolite production, methane and substrate degradability of polyphenol rich plant leaves and their component complete feed blocks. J Anim Sci Technol.

[26] Baruah L, Malik PK, Kolte AP, Dhali A, Bhatta R (2018). Methane mitigation potential of phyto-sources from Northeast India and their effect on rumen fermentation characteristics and protozoa *in vitro*. Vet World.

[27] Bhatta R, Saravanan M, Baruah L, Sampath KT, Prasad CS (2013). Effect of plant secondary compounds on *in vitro* methane, ammonia production and ruminal protozoa population. J Appl Microbiol.

[28] Bhatta R, Saravanan M, Baruah L, Sampath KT (2012). Nutrient content, *in vitro* ruminal fermentation characteristics and methane reduction potential of tropical tannin-containing leaves. J Sci Food Agric.

[29] Bhatta R, Baruah L, Saravanan M, Suresh KP, Sampath KT (2013). Effect of medicinal and aromatic plants on rumen fermentation, protozoa population and methanogenesis *in vitro*. J Anim Physiol Anim Nutr.

[30] Geneviève Z, Adama K, Balé B, Corrêa PS, Lemos LN, Vincent N (2018). *In vitro* rumen fermentation characteristics, methane production and rumen microbial community of two major *Acacia* species used in Sahelian region of Burkina Faso. Trop Subtrop Agroecosyst.

[31] Jadhav RV, Chaudhary LC, Agarwal N, Kala A, Kamra DN (2019). *In vitro* assessment of tropical tree leaves as modulators of rumen fermentation. Anim Nutr Feed Technol.

[32] Kaya E, Canbolat Ö, Atalay AI, Kurt O, Kamalak A (2016). Potential nutritive value and methane production of pods, seed and senescent leaves of *Gleditsia triacanthos* trees. Livest Res Rural Dev.

[33] Khelalfa K, Arhab R, Martín-García AI, Zaabat N, Belanche A (2020). Effect of *Acacia* purified tannins extract and polyethylene glycol treatment on *in vitro* ruminal fermentation pattern and methane production. Asia-Pac J Mol Biol Biotechnol.

[34] Mebirouk-Boudechiche L, Abidi S, Cherif M, Bouzouraa I (2015). Digestibilité *in vitro* et cinétique de fermentation des feuilles de cinq arbustes fourragers du nord est algérien. Rev Med Vet.

[35] Melesse A, Steingass H, Schollenberger M, Holstein J, Rodehutscord M (2019). Nutrient compositions and *in vitro* methane production profiles of leaves and whole pods of twelve tropical multipurpose tree species cultivated in Ethiopia. Agroforest Syst.

[36] Pal K, Patra AK, Sahoo A, Kumawat PK (2015). Evaluation of several tropical tree leaves for methane production potential, degradability and rumen fermentation *in vitro*. Livest Sci.

[37] Rira M, Morgavi DP, Popova M, Maxin G, Doreau M (2022). Microbial colonisation of tannin-rich tropical plants: Interplay between degradability, methane production and tannin disappearance in the rumen. Animal.

[38] Rira M, Morgavi DP, Archimède H, Marie-Magdeleine C, Popova M, Bousseboua H (2015). Potential of tannin-rich plants for modulating ruminal microbes and ruminal fermentation in sheep. J Anim Sci.

[39] Sallam SMA, Bueno ICS, Nasser MEA, Abdalla AL (2010). Effect of eucalyptus (*Eucalyptus citriodora*) fresh or residue leaves on methane emission *in vitro*. Ital J Anim Sci.

[40] Theart JJF, Hassen A, van Niekerk WA, Gemeda BS (2015). *In-vitro* screening of Kalahari browse species for rumen methane mitigation. Sci Agric.

[B41] Ángeles-Mayorga Y, Cen-Cen ER, Crosby-Galván MM, Ramírez-Bribiesca JE, Candelaria-Martínez B, Sánchez-Villarreal A (2022). *Foliage of tropical trees and shrubs and their secondary metabolites modify in vitro* ruminal fermentation, methane and gas production without a tight correlation with the microbiota. Animals.

[42] Brinkhaus AG, Wyss U, Arrigo Y, Girard M, Bee G, Zeitz JO (2017). *In vitro* ruminal fermentation characteristics and utilisable CP supply of sainfoin and birdsfoot trefoil silages and their mixtures with other legumes. Animal.

[43] Peng K, Xu Z, Nair J, Jin L, McAllister TA, Acharya S (2021). Conserving purple prairie clover (*Dalea purpurea* Vent.) as hay and silage had little effect on the efficacy of condensed tannins in modulating ruminal fermentation *in vitro*. J Sci Food Agric.

[44] Rajkumar K, Bhar R, Kannan A, Jadhav RV, Singh B, Mal G (2015). Effect of replacing oat fodder with fresh and chopped oak leaves on *in vitro* rumen fermentation, digestibility and metabolizable energy. Vet World.

[45] Soltan YA, Morsy AS, Sallam SMA, Lucas RC, Louvandini H, Kreuzer M (2013). Contribution of condensed tannins and mimosine to the methane mitigation caused by feeding *Leucaena leucocephala*. Arch Anim Nutr.

[46] Leitanthem VK, Bhar R, Kannan A, Khuman MW, Keshri A, Singh D (2019). Effect of replacement of oat fodder with fresh oak leaves on rumen fermentation and methane production in *in vitro* studies. J Entomol Zool Stud.

[47] SAS OnDemand for Academics [Internet] https://welcome.oda.sas.com/.

[48] Deeks JJ, Higgins JP, Altman DG, Higgins JPT, Thomas J, Chandler J, Cumpston M, Li T, Page MJ, on behalf of the Cochrane Statistical Methods Group (2019). Cochrane Handbook for Systematic Reviews of Interventions.

[49] St-Pierre NR (2001). Invited review: Integrating quantitative findings from multiple studies using mixed model methodology. J Dairy Sci.

[50] Souza VC, Almeida KV, Rosenstein PK, Desrues O, Panciroli N, Kebreab E (2025). Impact of tannin-based additives on animal performance and enteric methane emissions in dairy and beef cattle: A meta-analysis. J Dairy Sci.

[51] Iqbal N, Poór P (2025). Plant protection by tannins depends on defence-related phytohormones. J Plant Growth Regul.

[52] Mueller-Harvey I, Bee G, Dohme-Meier F, Hoste H, Karonen M, Kölliker R (2019). Benefits of condensed tannins in forage legumes fed to ruminants: Importance of structure, concentration, and diet composition. Crop Sci.

[53] Rufino-Moya PJ, Blanco M, Joy M (2021). Condensed tannins of sainfoin and their effect on ruminal fermentation. Influence of phenological stage and preservation. A review. ITEAAgrar Econ Tec Inf.

[54] Sakariyahu SK, McDowell T, Renaud JB, Papadopoulos Y, Glover K, Brown RN (2025). Profiling environmental variations in condensed tannins and other metabolites of birdsfoot trefoil (*Lotus corniculatus* L.) genotypes. Plants.

[55] Rana KK, Wadhwa M, Bakshi MPS (2006). Seasonal variations in tannin profile of tree leaves. Asian-Australas J Anim Sci.

[56] Makkar HPS (2003). Effects and fate of tannins in ruminant animals, adaptation to tannins, and strategies to overcome detrimental effects of feeding tannin-rich feeds. Small Ruminant Res.

[57] Rira M, Morgavi DP, Genestoux L, Djibiri S, Sekhri I, Doreau M (2019). Methanogenic potential of tropical feeds rich in hydrolyzable tannins. J Anim Sci.

[58] Kelln BM, Penner GB, Acharya SN, McAllister TA, Lardner HA (2021). Impact of condensed tannin-containing legumes on ruminal fermentation, nutrition, and performance in ruminants: A review. Can J Anim Sci.

[59] Huang XD, Liang JB, Tan HY, Yahya R, Ho YW (2011). Effects of *Leucaena* condensed tannins of differing molecular weights on *in vitro* CH4 production. Anim Feed Sci Technol.

[60] Horst EH, Ammar H, Khouja ML, Vargas JE, Andrés S, López S (2022). *In vitro* screening of the foliage of *Eucalyptus* species harvested in different seasons for modulating rumen fermentation and methane production. Agriculture.

[61] Thompson JP, Cristobal-Carballo O, Yan T, Lawther K, Dimonaco NJ, Zeller WE (2025). Unlocking the potential of willow condensed tannins: Effects on rumen fermentation, microbiome, and metabolome for sustainable ruminant nutrition. Anim Microbiome.

[62] Bueno ICS, Brandi RA, Fagundes GM, Benetel G, Muir JP (2020). The role of condensed tannins in the *in vitro* rumen fermentation kinetics in ruminant species: Feeding type involved?. Animals.

[63] Aboagye IA, Oba M, Castillo AR, Koenig KM, Iwaasa AD, Beauchemin KA (2018). Effects of hydrolyzable tannin with or without condensed tannin on methane emissions, nitrogen use, and performance of beef cattle fed a high-forage diet. J Anim Sci.

[64] Anantasook N, Wanapat M, Cherdthong A, Gunun P (2013). Changes of microbial population in the rumen of dairy steers as influenced by plant containing tannins and saponins and roughage to concentrate ratio. Asian-Australas J Anim Sci.

[65] Terra-Braga M, Poli CH, Tontini JF, Ahsin M, van Vliet S, Villalba JJ (2024). Trade-offs between selection of crude protein and tannins in growing lambs. J Anim Sci.

[66] Hindrichsen IK, Wettstein HR, Machmüller A, Soliva CR, Knudsen KEB, Madsen J (2004). Effects of feed carbohydrates with contrasting properties on rumen fermentation and methane release *in vitro*. Can J Anim Sci.

[67] Hindrichsen IK, Wettstein HR, Machmüller A, Jörg B, Kreuzer M (2005). Effect of the carbohydrate composition of feed concentratates on methane emission from dairy cows and their slurry. Environ Monit Assess.

[68] Sun ZQ, Li Y, Liu GB, Gao R, Bao JZ, Wang L (2022). Associative effects of ensiling mixtures of sweet sorghum and korshinsk pea shrub on fermentation quality, chemical composition, and *in vitro* rumen digestion characteristics. Anim Sci J.

[69] Van Soest PJ, Robertson JB, Hall MB, Barry MC (2020). Klason lignin is a nutritionally heterogeneous fraction unsuitable for the prediction of forage neutral-detergent fibre digestibility in ruminants. Br J Nutr.

[70] Jayanegara A, Togtokhbayar N, Makkar HPS, Becker K (2009). Tannins determined by various methods as predictors of methane production reduction potential of plants by an *in vitro* rumen fermentation system. Anim Feed Sci Technol.

[71] Van Soest PJ, Robertson JB, Hall MB, Barry MC (2020). Klason lignin is a nutritionally heterogeneous fraction unsuitable for the prediction of forage neutral-detergent fibre digestibility in ruminants. Br J Nutr.

[72] Niderkorn V, Baumont R (2009). Associative effects between forages on feed intake and digestion in ruminants. Animal.

[73] Knapp JR, Laur GL, Vadas PA, Weiss WP, Tricarico JM (2014). Invited review: Enteric methane in dairy cattle production: Quantifying the opportunities and impact of reducing emissions. J Dairy Sci.

[74] Hristov AN, Oh J, Firkins JL, Dijkstra J, Kebreab E, Waghorn G (2013). Special topics–Mitigation of methane and nitrous oxide emissions from animal operations: I. A review of enteric methane mitigation options. J Anim Sci.

